# Emerging strategies for the prediction of behaviour, growth, and treatment response in vestibular schwannoma

**DOI:** 10.1007/s00701-025-06522-7

**Published:** 2025-04-22

**Authors:** Daniel Lewis, Ka-loh Li, Ibrahim Djoukhadar, Cathal J. Hannan, Omar N. Pathmanaban, David J. Coope, Andrew T. King

**Affiliations:** 1 Division of Cancer Sciences, School of Health Sciences, Faculty of Biology, Medicine and Health, University of Manchester, Manchester, UK; 2 Geoffrey Jefferson Brain Research Centre, University of Manchester, Manchester, UK; 3 Division of Informatics, Imaging and Data Sciences, School of Health Sciences, Faculty of Biology, Medicine and Health, University of Manchester, Manchester, UK; 4 Division of Neuroscience, School of Biological Sciences, Faculty of Biology, Medicine and Health, University of Manchester, Manchester, UK; 5 Division of Cell Matrix Biology & Regenerative Medicine, School of Biological Sciences, Faculty of Biology Medicine and Health, University of Manchester, Manchester, UK; 6 Department of Neurosurgery, Salford Royal Hospital, Nothern Care Alliance NHS Foundation Trust, Manchester, M6 8HD UK

**Keywords:** Biomarkers, DCE-MRI, Inflammation, NF2-SWN, Radiomics, Vestibular schwannoma

## Abstract

Vestibular schwannoma (VS) can present several management challenges for the clinician. Their unpredictable potential for growth creates uncertainty regarding when active treatment should be initiated, and once growth is confirmed which treatment option should be adopted, notably surgery or radiotherapy, and in particular stereotactic radiosurgery (SRS). The obvious benefits of SRS would ideally come with the ability to reliably predict long-term radiosurgery response/failure. Differentiation from temporary post-treatment phenomena such as transient tumour expansion or reactive swelling remains an unmet need. More powerful again would be the pre-treatment identification of which tumours will respond to radiosurgery and which will not. Over the past decade, there has been emerging interest in the development of non-invasive biomarkers, including imaging, for predicting growth and treatment response in VS. Alongside clinical radiographic predictors for VS growth such as extracanalicular tumour location and growth in the first year, studies have shown potential promise for advanced MRI and blood-based biomarkers that capture pathophysiological mechanism behind VS growth. Emerging interest in *radiomics*-based analyses of routinely acquired MRI, and the use of physiological imaging techniques such as dynamic-contrast enhanced MRI for pre- and post-treatment evaluation of tumour microvasculature and microstructure holds promise for revolutionizing this area. This article explores the current state of identifying VS growth at initial presentation, predicting treatment response to SRS and detecting early treatment failure, and finally the potential for developing more personalized patient selection for drug therapies, including bevacizumab, as well as emerging novel therapeutics for these tumours.

**Supplementary Information:**

The online version contains supplementary material available at 10.1007/s00701-025-06522-7.

## Introduction: The challenge of vestibular schwannoma behaviour

Vestibular schwannomas (VS) are histologically benign tumours that originate from the myelin-forming Schwann cell layer surrounding the vestibular portion of the vestibulocochlear nerve [1, 33]. Their lifetime incidence approximates 1 in 1000, accounting for over 8% of intracranial tumours in adults [33]. In approximately 95% of cases VS occur as a unilateral, sporadic entity but they can also occur as part of the dominantly inherited tumour syndrome NF2-related schwannomatosis (NF2-SWN) where they commonly present as bilateral multifocal tumours afflicting both nerves, or less commonly unilaterally as part of *LZTR1-*related schwannomatosis [63]. These tumours most commonly present initially with otological and/or vestibular symptoms but as tumours grow larger, adjacent cranial nerve and brainstem structures can be compressed producing significant morbidity and eventually a risk to life [33].

One key challenge in VS management is their unpredictable natural history [46]. Up to 70% of untreated tumours demonstrate some volumetric growth at 3 years [41, 44, 59] with clinically relevant growth seen in up to 30 to 50% of VS at 5 years after diagnosis on linear measurements [44, 56, 62]. For larger or growing sporadic tumours, treatment either with surgical excision or stereotactic radiosurgery (SRS) is recommended [21, 33]. Considerable debate remains though as to the size at which active treatment should be initiated and which patients should be selected for SRS rather than surgery. The multifocal and bilateral nature of NF2-SWN VSs present a particular management challenge, and inferior outcomes through surgery or radiotherapy are seen compared to sporadic tumours [7]. Whilst the anti-angiogenic agent bevacizumab has a proven role against progressive NF2-SWN VSs, there is a recognised cohort of patients not responding to treatment and significant concerns about treatment response duration and complications of long-term bevacizumab use [7, 51]. What is required for encountering these challenges, are better biomarkers than can help us to not only understand and predict VS growth but also select patients for treatment, and in this review we will cover some of the established and emerging biomarkers in this field.

## Predicting VS growth

An essential factor in clinical decision-making for VSs is expected growth. Being able to predict VS growth soon after diagnosis would enable better monitoring and earlier intervention. Most research around predictors of VS growth has focused on predictive patient demographics, presenting symptoms, and tumour factors such as size, location, and growth pattern (Supplementary Table S1). Younger patient age [67, 68] and biological sex [58] might influence VS growth, but there remains considerable debate regarding such associations [23, 40, 46]. The association between tinnitus, presentation hearing loss and later growth also remains uncertain [46, 62, 80]. Possibly reflecting the vestibular nerve origin of these tumours, there is, however, stronger evidence in support of a putative link between imbalance/dysequilibrium symptoms at diagnosis and later growth, [23, 80]. Amongst the tumour related factors, larger tumour size [23, 41, 68] and extracanalicular location [19, 56, 61, 68] have been shown to be predictors of later growth, with a recent meta-analysis of 12 studies and 6198 patients [80] identifying tumour location, initial size, cystic change, and vestibular symptoms as possible growth determinants.

VSs identified to be growing are likely to continue to grow, and Sethi et al. [61] used conditional probability and the a priori information of a tumour’s growth behaviour, to demonstrate that the longer a VS has been stable, the lower the overall risk of future growth in subsequent years. An association between macrocystic change and VS growth [27, 67, 68, 73] has also been reported and intratumoural haemorrhage may also play a role in cystic change and rapid growth (> 4 mm/year) of some tumours [46]. One study demonstrated that the proportion of cystic tumours that grew was 75% vs 40% in non-cystic VS [68], and in this study cystic change alongside younger age, extracanalicular extent, larger volume, and growth during 1^st^ year were included in a scoring system with demonstrable accuracy, sensitivity and specificity for later growth prediction.

### Emerging mechanistic biomarkers of VS behavior

**Fig. 1:**
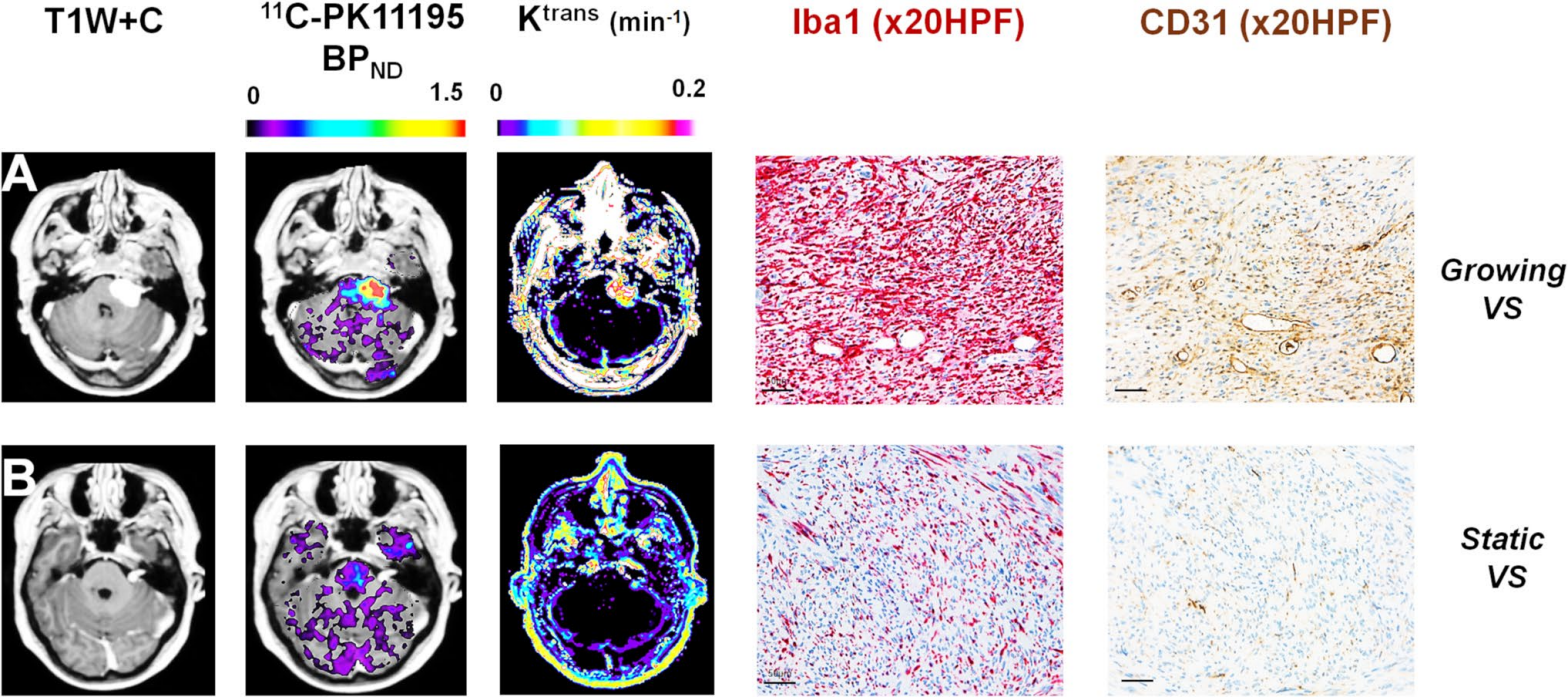
Imaging of intratumoural inflammation in growing VS. Representative imaging and histology from a patient with a growing VS (**A**); and a static VS (**B**) is shown. From left to right: axial T1W post-contrast images; parametric map of ^11^C-PK11195 PET specific binding (BP _ND_) overlaid on MRI; map of DCE-MRI derived K^trans^; and immunostains demonstrating Iba1 (Iba1 red, immunoperoxidase) TAM density and microvessels (CD31 *brown,* immunoperoxidase) in each tumour (20 × high-powered field, HPF). Within the growing VS there is higher binding of the inflammation PET tracer, ^11^C-PK11195, and elevated K ^trans^ suggesting indicating increased vascular density/permeability compared with the static tumour. Comparative immunohistochemistry (Iba1 red, immunoperoxidase) demonstrates an abundance of intratumoural Iba1 ^+^ macrophages and corresponding regions of high vascularity within the growing tumour

One challenge in appraising clinical predictors of VS growth is variability in how growth is defined and measured (e.g. linear vs volumetric) [46, 68, 80], and further large-centre studies/meta-analyses on this topic with uniform methodology are welcome. An area, however, that may also hold promise for growth prediction are emerging ‘mechanistic’ or ‘physiological’ biomarkers that provide an insight into pathophysiological process such as inflammation and angiogenesis that may drive VS growth. Tumour associated macrophages (TAM) are near ubiquitously found within Antoni type B areas within VS [21, 33] and alongside other immune cells (e.g. T cells) they constitute over 50% of the cellular content within sporadic and NF2-SWN associated tumours [17, 22, 26, 33, 34]. TAM presence correlates with tumour size [21, 22, 33] and VS growth rate [22, 33, 34] but also poorer hearing [18, 21, 57], and growth following subtotal resection [48]. Directly quantifying TAM within a given VS may therefore carry predictive growth potential and in the first in vivo exploration of this it was demonstrated that compared to static tumours, growing VS displayed higher specific binding of a TSPO (translocator protein) PET tracer for inflammation [33]; with the source of this binding being an abundance of TSPO-expressing TAM (Fig. 1). In another PET study, Anton-Rodriguez et al. [3] evaluated uptake of the metabolic PET tracer ^18^F-FDG and the cellular proliferation marker ^18^F-FLT (3’-deoxy- 3’-fluorothymidine) in six patients with NF2-SWN associated VS, demonstrating significantly higher uptake in rapidly growing VSs compared to indolent or slow growing tumours. The cellular source of the observed ^18^F-FDG and ^18^F-FLT uptake in these tumours was not confirmed, but given the prominent infiltration of proliferating Iba1^+^ macrophages seen in growing tumours [17, 26, 33, 34], they can be hypothesised as a principal cellular origin of his uptake.

Given its more widespread availability and lower cost, the ideal approach for predicting VS growth would be a specific MRI derived biomarker. Earlier, small studies demonstrated that *radiomic-based* analyses of contrast-enhanced T1 weighted imaging [25] and use of normalised relative [78] signal intensity values could potentially differentiate VS growth groups. The most recent exciting developments, however, come from use of advanced physiological MR imaging techniques, such as dynamic contrast enhanced MRI (DCE-MRI) [12, 24, 33, 34, 45]. Through measuring and modelling the pharmacokinetics of intravenously administered gadolinium-based contrast agents DCE-MRI allows non-invasively quantification of tissue microvascular structure [33, 34, 36, 37]. Lewis et al. demonstrated that growing VS displayed significantly higher tumour K^trans^, a DCE-MRI derived measure of vascular permeability and microvascular flow [33, 71], with K^trans^ values correlating with tissue markers of microvessel density, tumoural TSPO PET, and tissue TAM density (Fig. 1). A subsequent comparative study of diffusion-weighted imaging (DWI) and DCE-MRI metrics in NF2-SWN and sporadic tumours furthermore demonstrated that both VS groups show size and pre-treatment growth dependant increases in tumour K^trans^, the extravascular-extracellular space fraction (v_e_) and tumoural free water content [34].

In these earlier studies, patients were imaged at variable timepoints and crucially after the natural growth history of a given VS had already been established. Schouten et al. [69], however, in a clear demonstration of the potential predictive power of these techniques undertook a large DCE-MRI study on 110 patients with newly diagnosed sporadic VS. The authors showed a highly significant correlation between DCE-MRI derived microvascular kinetic parameters and subsequent VS growth at 1 year, with high baseline tumour K^trans^ values(> 0.16 min^−1^) being highly predictive of future growth (OR 15.6); and with the combination of K^trans^ and v_e_ providing an internally validated growth prediction model with high sensitivity and specificity. Larger multi-centre studies with inclusion of smaller intracanalicular VS are required to better understand the reproducibility of these parameters and their application across the wider VS population, but this important study and earlier work highlights the potential promise of these physiological imaging techniques in elucidating VS growth potential.

### The road towards combined predictive panels for VS growth potential

There is growing evidence that despite the non-invasive nature of VS, these tumours can exert widespread effects remote to the tumour [1, 22, 28, 74]. Alongside predictive imaging biomarkers of growth, there has also been interest in other non-invasive metrics such as peripheral blood testing. In an early study, Kontorinis et al. demonstrated that neutrophil-to-lymphocyte ratio (NLR) values, a potential marker of subclinical inflammation, were higher in patients with growing VS compared to compared to static controls [28]. Other groups have similarly found decreased progression-free survival in patients with high baseline serum C-reactive protein at VS diagnosis [74]. The cause of the apparent systemic inflammation seen is incompletely understood but tumoural induction of vascular endothelial growth factor (VEGF), interleukin- 1β (IL- 1β), and interleukin- 6 (IL- 6) secretion have all been implicated [8, 10]. Recent studies have shown upregulation of several immune-related biomarkers in the plasma of patients with VS [22, 70], in keeping with the inflammatory nature of these tumours, with Hannan et al. finding significantly increased concentrations of several monocytic chemokines in patients with established growing tumours compared to a non-growing tumour cohort [22]. Within the CSF some studies have similarly demonstrated higher concentrations of immunomodulatory cytokine CCL2 and CCL18 in the CSF of patients with larger volume tumours [6]. Larger studies that assess the prospective potential of these blood and CSF based biomarker, their repeatability and reproducibility, and their sensitivity to dynamic changes in tumour size and growth are clearly required. Such studies, however, build upon evidence that immune cell infiltration is a relevant driver of growth in VS [17, 22], and highlight potential for combined panels of both clinical features and non-invasive imaging and blood-based biomarkers for VS growth prediction in the near future.

## Predicting VS response to radiosurgery

Although stereotactic radiosurgery is an established, effective treatment for patients with small- to moderate-sized vestibular schwannomas (VS) [20] key challenges in the use of SRS remain such as: the unpredictable time to tumour response; the recognised 10—15% of tumours that show long-term tumour growth/treatment failure [55]; and the phenomenon of transient tumour expansion (TTE) or reactive swelling that can occur in up to 63% of SRS treated patients and up to 5 years after irradiation [4, 55]. There is currently no way of discriminating this reactive swelling from continued growth or treatment failure. This inability to differentiate these different responses often leads to delayed recognition of treatment failure, with the consequent challenges of dealing with both a significantly larger tumour and the potential increased technical difficulties of surgery due to prior irradiation [35, 75]. The establishment of in vivo biomarkers that can both predict which tumours will respond to SRS but also identify non-responding VS early on following treatment, would therefore be of considerable benefit in stratifying patients that require closer radiological surveillance and/or early surgical resection.

Previous studies have identified factors derived from routine clinical MRI sequences that can predict VS SRS response. Larger tumours are known in general to have a decreased overall tumour control rate [13], although large recent studies have suggested that even for patients with Koos grade IV and tumours > 3 cm, SRS can still be an effective primary treatment option for selected patients [49]. Higher pre-treatment growth rate can also portend to poorer outcomes from SRS [42], with fast growing tumours in one study (defined as a volume doubling time less than 15 months) showing 10 year control rates of only 67.6% compared to 86.0% in slower growing tumours [30]. The impact of macrocystic change on SRS outcome remains debated in the literature. The prior belief that cystic tumours do not respond well to SRS is increasingly challenged [11, 77], with one study of 187 patients demonstrating that a pre-SRS cystic component was in fact associated with greater volume reduction [77]. The role of routine DWI in predicting SRS response is similarly debated. Some studies of solid VS have shown higher pre-treatment ADC in patients with tumour regression or stabilization post SRS compared to those with progression [15, 65, 77]. Other studies conversely show that the pretreatment ADC is lower in responders [12], although in this particular study half of included tumours had FU < 3.5 years. Across studies there is heterogeneity in sample size, length of follow-up, DWI measure used, and definition of tumour response, and there is clearly a need for larger studies in this area with standardized analysis approach, patient selection, and outcome definition.

One emerging and exciting use of clinical MRI for image based prediction of SRS response, is the use of radiomic-based or texture analysis approaches [66, 79] (Supplementary Table S2). MRI textural differences such as the grey-level inhomogeneity can reflect variations in tumour biology [16, 31, 32] and may impact on treatment response. Early small studies demonstrated that radiomic features extracted from T1-weighted, T2-weighted and post-contrast imaging such as grey-level matrices and kurtosis of relative T2-weighted image intensity values could be used to differentiate responders but these studies were limited by a lack of clear definition of what constitutes treatment response/treatment failure, and the failure to account for potential early/transient progression in some tumours within the short follow-up period. In a larger study by Langenhuizen et al. [32], 85 tumours undergoing SRS were included for tumour texture analysis. Using grey-level co-occurrence matrices (GLCM: a texture analysis method that calculates how often pixels with specific values occur in an image and their spatial relationship), and strict criteria for long-term tumour control and tumour progression, they derived a model with 83% accuracy for treatment outcome, with improved accuracy for increasing tumour volumes (especially tumours > 5 cm^3^). In another work, it was shown that a set of four GLCM features, achieved a sensitivity of 0.82 and a specificity of 0.69 for predicting TTE, with similarly greater prediction in larger tumours (volume > 6 cm^3^) [31]. In one of the largest studies to date Yang et al. [79] used a two-level machine-learning model to predict both the long-term outcome and the occurrence of TTE after SRS. Using five radiomic features that capture T2-weighted intensity and contrast enhancement inhomogeneity, accuracy of long-term outcome and TTE prediction was 88.4% (AUC = 0.913) and 85.0% (AUC = 0.881) respectively.

### Emerging role of DCE-MRI in predicting VS SRS response

One obstacle in accurately predicting and evaluating VS radiosurgery response is our incomplete understanding of the biological mechanisms that underlie post-SRS growth arrest [35, 64]. Conventional fractionated radiotherapy preferentially targets rapidly dividing cells in the M and G_2_ phase of the cell cycle, relying on direct cell killing and loss of cell reproductive ability due to DNA damage. The therapeutic effect of high dose (> 10 Gy) radiotherapy such as SRS, by contrast, may depend on both direct DNA-mediated cell toxicity and other indirect effects such as potentiation of anti-tumour immune responses and radiation-induced vascular damage to immature tumour blood vessels [35, 47, 64]. There has been recent interest in using advanced MRI techniques such as DCE-MRI to evaluate these possible indirect radiosurgery effects and microvascular changes in the post-SRS VS microenvironment (Supplementary Table S3). In one small pilot study, five patients underwent imaging pre-treatment and up to 6 months post radiosurgery using a high-spatial resolution DCE-MRI acquisition. Changes in tumour microvascular parameters such as K^trans^ were evident at 2 weeks post-treatment, with a marked decrease in DCE-MRI derived parameters at 6 months post-treatment in all tumours [35]. A sodium (^23^Na) MRI acquisition for quantifying changes in tumour tissue sodium concentration (TSC) was also adopted, demonstrating that post-treatment tumour TSC increased up to 6 months, with tumour TSC changes preceding changes in tumoural diffusion metrics and tumour size. Early radiation-induced endothelial damage and increased capillary permeability may underlie the early increases in K^trans^, v_e_ and TSC seen in this study, whereas at later time points fibrinoid degeneration of the vascular walls, as well as endothelial and pericyte cell proliferation can occur, abrogating in vivo perfusion metrics [2, 35]. In a larger study of 24 VS undergoing SRS, Ozer et al. similarly demonstrated lower *K*^trans^ and *V*_e_ values at three and 6 months after radiosurgery compared to pre-treatment in patients with regression or TTE, but not ongoing tumour enlargement at 48 months [45]. Most recently Meng et al. [43] used golden-angle radial sparse parallel (GRASP) MRI and semi-quantitative DCE parameters such as peak, area under the curve (AUC), and wash-in slope to characterize the vascular permeability changes in 19 post-SRS VS. Only cases with transient loss of central homogenous contrast enhancement, a possible marker of early tumour vascularity reduction, were included. At 6 months all semi-quantitative parameters were significantly reduced relative to pre-treatment; with peak, AUC and wash-in slope values remaining significantly decreased at 30 months.

**Fig. 2:**
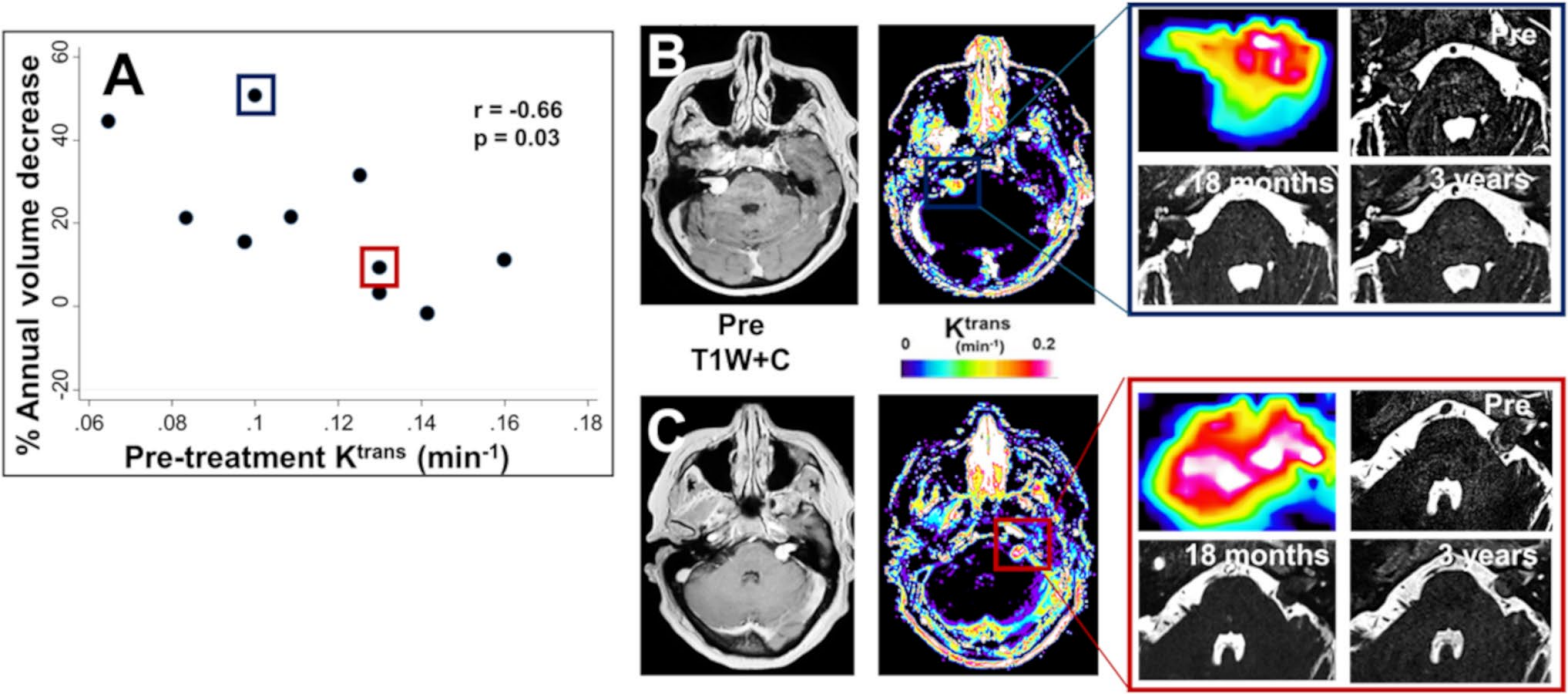
Tumour microvascular metrics as a potential predictor of VS SRS response. **A** Intertumour scatterplot demonstrating negative association between pre-treatment tumour K^trans^ and % volume reduction at 18 months post SRS. Pearson’s product-moment correlation coefficient (r) reported. **B** Representative patient with a right sided VS and pre-treatment tumour K^trans^ of 0.10 min^−1^ (*blue box in panel A*). At 18 months and 3 years post-treatment there was a dramatic reduction in tumour size with a 77% and 79% volume reduction respectively on follow up high-spatial resolution T2-weighted imaging. **C** Representative patient with a left sided VS and a pre-treatment tumour K^trans^ of 0.13 min^−1^. (*red box in panel A*) Compared to the tumour shown in panel B there was a much smaller volume reduction at 18 months (15%) and 3 years (30%) post treatment on follow up high-spatial resolution T2-weighted imaging

Following treatment, the ability to differentiate early between tumours that are responding to SRS would allow closer monitoring and hopefully more timely rescue surgery before significant tumour regrowth or post-radiosurgery scarring occurs. The ‘holy grail’, however, is a clinically applicable accurate biomarker that can predict response pre-treatment, allowing selection of patients most likely respond to SRS. In a study of 35 patients, Hwang et al. [24] demonstrated differences in baseline/pre-treatment DCE-MRI derived parameters between responding (defined as tumour volume was reduced by more than 20% on the last follow-up MRI) and non-responding tumours. Responding tumours demonstrated significantly lower mean pre-treatment tumour K^trans^ and area under the DCE curve of the initial 90-s postcontrast agent arrival (IAUC_90_), with *K*^trans^ and IAUC_90_ showing a respective sensitivity/specificity of 81.8%/69.2% and 100%/53.8% for tumour response prediction. It has been postulated that a higher pre-treatment K^trans^ might reflect greater pre-treatment growth, macrophage infiltration and angiogenesis, and that in such tumours vascular damage incurred by a single 13 Gy dose may be insufficient to attenuate tumour vasculature and growth (Fig. 2). Larger studies with at least 5 years follow up post radiosurgery and with standardized analysis approaches incorporating both quantitative and semi-quantitative parameters that do not require vascular deconvolution are required to better understand the predictive potential of DCE-MRI.

## Predicting VS response to drug therapy

Beyond radiosurgery, there is growing interest the use of targeted medical therapies for treating VS that prioritize both tumour control and hearing preservation, and this need for new medical therapies is especially profound in NF2-SWN associated VS. An important consideration, however, is determining the optimal biomarkers for patient selection into clinical trials of such therapies, and then monitoring disease response during treatment. Indeed, the ideal biomarkers would display not only high precision and sensitivity for treatment related changes but also high repeatability, reproducibility, and applicability across multiple centres.

### Biomarkers of anti-angiogenic (Bevacizumab) therapy

**Fig. 3:**
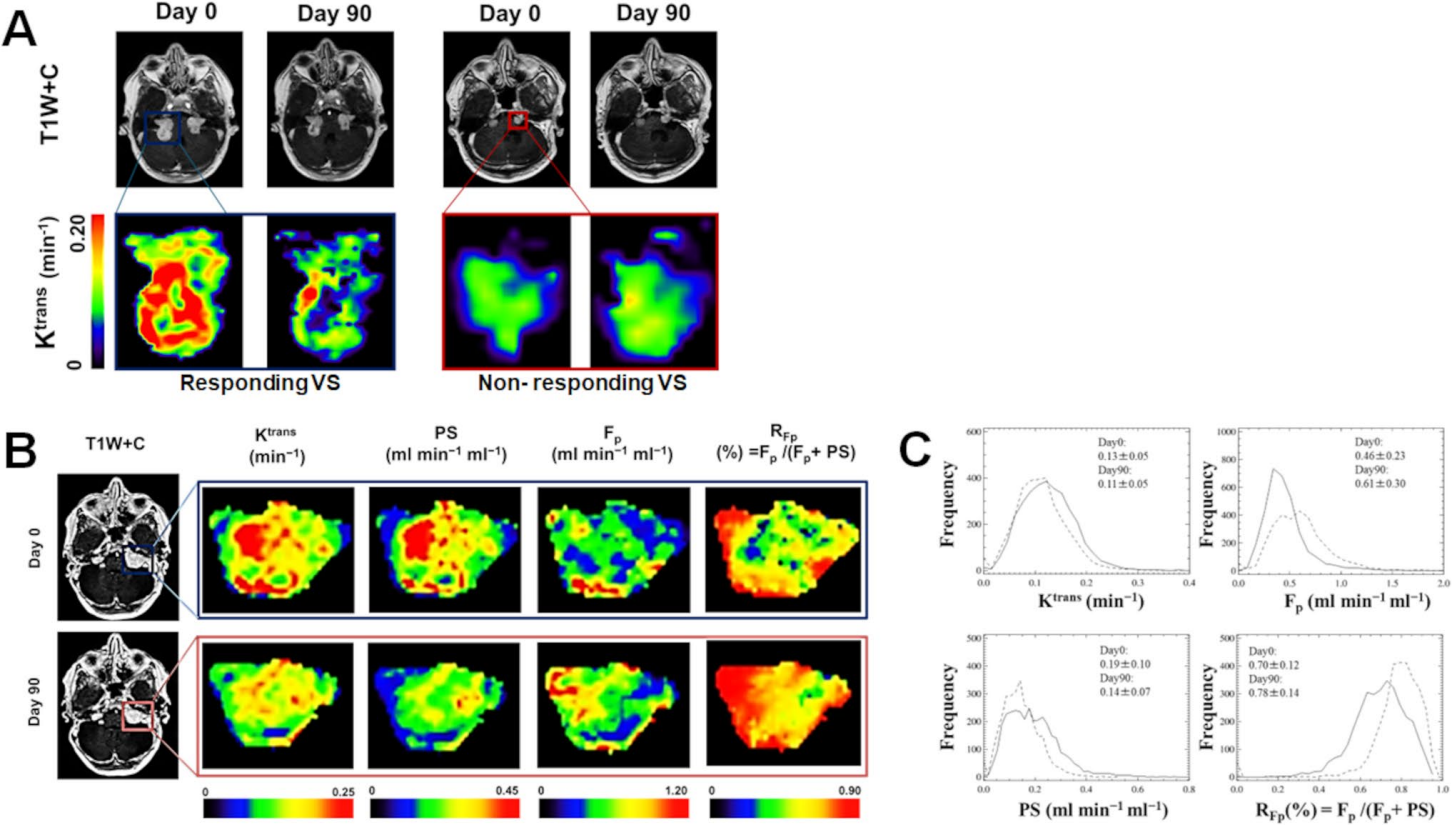
Biomarkers of anti-angiogenic (Bevacizumab) response in NF2-SWN associated VS. **A** Representative axial T1 W post-contrast images (*top row*) and DCE-MRI derived K^trans^ maps (*bottom row*) at pre-treatment (day 0) and at 3 months post 5 mg/kg bevacizumab therapy. Maps for index treated tumour shown. Left panels shows a patient with bilateral responsive VS that decreased in size following treatment, right panels shows a patient with bilateral non-responsive VS. Note that bevacizumab responsive tumours had higher pre-treatment (baseline) K^trans^ than non-responding tumours and showed reductions in K^trans^ and tumour volume at 90 days into therapy. **B** Maps of DCE-MRI derived tumour transfer constant (K^trans^), the capillary permeability-surface area product (PS), capillary plasma flow (F_p_), and the ratio (R_Fp_) of plasma flow to the sum of F_p_ and PS at day 0 (baseline) and 3-months (post-treatment) in a different patient undergoing bevacizumab treatment. Decreases in VS volume, K^trans^ and PS are seen post-treatment, with a corresponding increase in RFp and the tumour subregion displaying unchanged/increased perfusion but inadequate permeability. **C** Histograms of tumour voxel values for K^trans^, F_p_, PS and R_Fp_ at baseline (solid line) and 3-months post-treatment (dashed line), for the same VS shown in panel B. Tumour mean ± SD are shown

The only current adopted medical therapy against VS, is the anti-VEGF antibody bevacizumab (Avastin©). Tumour volumetric response rates of 40–60% and hearing response rates of ~ 45% are seen, but responses can be varied and long-term bevacizumab use is associated with high rates of significant adverse events such as hypertension, proteinuria, and renal impairment [8, 14, 51, 52]. The early seminal trials of bevacizumab similarly found that high pre-treatment tumour ADC (a diffusion correlate of tumour free fluid) correlated with changing tumour volume at 3 months and that treatment-induced ADC decreases were associated with hearing response [52]. In a subsequent longitudinal DCE-MRI study, Li et al. [36] demonstrated that compared to non-responders, responding NF2-SWN VS (defined as a volume reduction exceeding 0.125 cm^3^ or a relative volume decrease exceeding 5% after 90 days 5 mg/kg bevacizumab therapy) had higher pre-treatment K^trans^ and lower R1_N_. At 90 days responders showed increases in R1_N_ and reductions in K^trans^ and ADC in association with volume loss; effects hypothesised to represent vascular normalization and reductions in interstitial tumour fluid [34, 60] (Fig. 3A).

Although the primary action of anti-VEGF therapy is thought to be rapid, early reduction in capillary endothelial membrane permeability [50, 76], changes in tumour K^trans^ could also reflect post-treatment changes in regional capillary blood flow [71]. To better elucidate this, Li et al. used multi-kinetic modelling to capture high-spatial resolution treatment-related changes in microvascular flow and permeability within the VS microenvironment [38]. They demonstrated that K^trans^ changes following bevacizumab treatment are primarily driven by a reduction in capillary endothelial membrane permeability, rather than changes in tumour microvascular blood flow (Fig. 3B, C), with a multivariate model combining pre-treatment v_e_, the capillary permeability-surface area product (PS), and capillary flow demonstrating high sensitivity (83%) and specificity (87.5%) for prediction of VS volumetric response at 90 days.

There has also been interest in combined imaging serum biomarker panels for predicting bevacizumab response. Blakeley et al. [8] studied changes in MRI-derived biomarkers (ADC, K^trans^) and circulating biomarkers in 14 patients undergoing higher dose Bevacizumab 7.5 mg/kg therapy. In line with the findings of Li et al. [36], they demonstrated that radiological response (≥ 20% volume decrease at 6 months) but not hearing response was associated with higher pre-treatment tumour K^trans^ and reductions in both plasma free VEGF/VEGF-D and the Ang2/Tie- 2 axis, as measured through soluble Angiopoietin- 1 receptor (sTie- 2) [9]. In contrast to low-dose regimens, a study of high-dose bevacizumab therapy (10 mg/kg) demonstrated that plasma levels of VEGF did not significantly decrease during treatment, although increases in plasma levels of other cytokines associated with abrogation of the VEGF axis were seen [5]. Significantly higher levels of free VEGF at week 3 during treatment were seen in the included paediatric population though, who displayed lower radiological and hearing response rates overall [5]. Further studies are clearly required to better understand the dynamic changes and predictive potential of circulating biomarkers such as free plasma VEGF and sTie- 2 during anti-angiogenic therapy. Alongside the aforementioned imaging profile on pre- and post-treatment MRI, however, these findings do suggest promise for finding sensitive blood biomarkers of anti-VEGF therapy resistance and justify their exploration in future studies [53].

### Emerging medical therapies against VS and their implication for biomarker development

Beyond bevacizumab other therapies trialled against VS include mTOR inhibitors such as everolimus, tyrosine kinase inhibitors, and a VEGF receptor vaccine. To date though these drugs have shown either inferior hearing/radiographic response rates or unacceptable rates of adverse events [14]. Most recently a phase II platform trial of once daily brigatinib (an inhibitor of ALK and multiple other tyrosine kinases) demonstrated hearing improvement (35% of ears) and radiographic response (10% of target tumours), with the greatest responses seen for meningiomas and non-vestibular schwannomas [54]. In non-small cell lung cancer, mutations of the anaplastic lymphoma kinase (ALK) gene are predictive biomarkers for response to ALK inhibitors like brigatinib. To date though no reliable invasive or non-invasive markers of predicting response to ALK small-molecule tyrosine kinase inhibitors (TKIs) in NF2-SWN associated tumours have been found.

Given our emerging understanding of the role that the inflammatory microenvironment plays in VS pathobiology and growth, there is also considerable interest in early-phase immunotherapy trials. A currently recruiting phase-II clinical trial is randomizing patients to 325-mg aspirin twice daily, or placebo, with tumour growth the outcome measure (NCT03079999), and there has also been preliminary investigation of immune checkpoint inhibition strategies targeted against the immunosuppressive activity of the PD-L1/PD- 1 axis [29]. Our understanding of PD-L1/PD- 1 axis blockade in VS is at a primitive stage but its potential is highlighted in a single case report where growth arrest of a sub-totally resected VS post SRS was achieved for 30 months following pembrolizumab treatment [29]. With the potential dawn of ‘VS immunotherapy’, reproducible and widely applicable biomarkers for quantifying changes in immune cell populations in VS will be needed. Given the link between inflammation and angiogenesis in VS [22, 33, 34], and the possible potential for augmenting immunotherapy response in VS through anti-VEGF vascular normalization [39], the aforementioned imaging and blood biomarkers of tumour microvasculature could play a role here. There is also a need for more specific biomarkers that can directly quantify the immune cell population in vivo, and novel biomarkers which are currently being investigated in VS include contrast agents such as ultrasmall superparamagnetic iron oxide nanoparticles or USPIO (NCT06572475). After extravasation these nanoparticles are thought to be rapidly phagocytosed by TAM and on delayed (> 24 h) MRI this engulfment and focal concentration of USPIO within TAM can be detected and quantified [72]. Further data is needed on the sensitivity of such biomarkers to treatment-induced changes in immune cell abundance and phenotype, and through parallel incorporation in new immunotherapy trials such biomarkers can be robustly assessed.

## Conclusions

Robust prediction of future VS growth and response to established treatments such as SRS and anti-VEGF therapy in NF2-SWN associated VS remains an unmet need. Through leveraging of advanced imaging, artificial intelligence, and other non-invasive methods we have begun to not only gain a greater understanding of VS pathophysiology but also develop biomarkers with good sensitivity and specificity for predicting growth and response to established therapies. Larger scale studies with standardised methodology and robust uniform definitions of what defines tumour growth and treatment success are required to move promising early-stage biomarkers along the translational roadmap and evaluate their precision, multicentre reproducibility, and wider clinical applicability. The emergence of trials of novel therapeutics against these tumours also presents a unique opportunity to progress this field further, and ultimately bring better, more personalised treatment decisions to our patients.

## Supplementary Information

Below is the link to the electronic supplementary material.

Supplementary file1 (DOCX 47 KB)

## Data Availability

No datasets were generated or analysed during the current study.
