# Supplementary material for: Emerging strategies for the prediction of behaviour, growth, and treatment response in vestibular schwannoma
Source: Acta Neurochir (Wien). 2025 Apr 22;167(1):116. doi: 10.1007/s00701-025-06522-7 (PMC12014738; doi:10.1007/s00701-025-06522-7)
Supplement: Supplementary file 1 — Supplementary file1 (DOCX 47 KB) [file 701_2025_6522_MOESM1_ESM.docx]

**Supplementary Table S1: Reported predictive clinical/ radiographic factors associated with VS growth**

| **Factor** | | **Studies reporting statistical difference**  **(included number of patients in each study)** | **Studies reporting no difference**  **(included number of patients in each study)** |
| --- | --- | --- | --- |
| **Patient factors** | | | |
| **Younger patient age** | | Tang 2014 (88)  Patniak et al, 2015 (576)  Prasad 2018 (576)  Nilsen 2020 (204)  Stastna 2022 (125)  Stastna 2024 (615) | Flint 2005 (100)  Herwadkar 2005 (50)  Whitehouse 2010 (88)  Paldor 2016 (2784 patients across 22 included studies in review)  Daultrey 2016 (900)  Hunter et al 2016 (564)  Van linge 2016 (155)  Younes 2017 (53)  Borsetto 2018 (112)  Lees 2018 (361)  D’Haese 2019 (62)  Fieux 2020 (336)  Kleijwegt 2020 (738)  Schnurman 2020 (212)  Higuchi 2021 (53)  Kim 2021 (118)  Sethi 2021 (340)  Marinelli 2022 (952)  Itoyama 2022 (64)  Yamada 2022 (31)  Truong 2023 (78)  Yagi 2023 (67)  Marinelli 2023 (405) |
| **Biological sex** | | Sakamoto 2001 (31) | Herwadkar 2005 (50)  Paldor 2016 (2847 patients across 18 included studies in review)  Daultrey 2016 (900)  Hunter et al 2016 (564)  Younes 2017 (53)  Borsetto 2018 (112)  Lees 2018 (361)  D’Haese 2019 (62)  Fieux 2020 (336)  Kleijwegt 2020 (738)  Kim 2021 (118)  Sethi 2021 (340)  Yamada 2022 (31)  Truong 2023 (78)  Yagi 2023 (67) |
| **Presenting symptoms** | **Tinnitus at presentation** | Agrawal 2010 (180)  Timmer 2011 (240)  Breivik 2012 (186)  Hentschel 2020 (1217) | Paldor 2016 (331 patients across 4 included studies in review)  Lees 2018 (361)  D’Haese 2019 (62)  Sethi 2020 (340)  Yagi 2023 (67) |
|  | **Hearing loss at presentation** | Tschudi 2000 (74, * hearing loss a predictor of lower tumour growth compared to tinnitus, sudden hearing loss and dizziness)  Fieux 2020 (336,* hearing loss associated with reduced risk of fast VS growth)  Truong 2023 (78, *hearing loss < 2 years was predictor of growth) | Flint 2005 (100)  Hoistad 2001 (102)  Godefroy,2009 (70)  Whitehouse 2010 (88)  Agrawal 2010 (180)  van de Langenberg 2011 (36)  Varughese 2012 (178)  Hunter et al 2016 (564)  Lees 2018 (361)  D’Haese 2019 (62)  Sethi 2020 (340) |
|  | **Imbalance and disequilibrium (*not vertigo)** | Artz 2009 (234)  Malhotra 2009 (202)  Timmer 2011 (240)  Breivik 2012 (186)  Jethenamest 2015 (91)  Hunter 2016 (564)  Wolbers 2016 (155)  Fieux 2020 (336)  Hentschel 2020 (1217)  Nilsen 2020 (204)  Dardis 2022 (443) | Hoistad 2001 (102)  Godefroy,2009 (70)  Agrawal 2010 (180)  van de Langenberg 2011 (36)  Varughese 2012 (178)  D’Haese 2019 (62)  Sethi 2020 (340)  Higuchi 2021 (53) |
|  | **Vertigo** |  | Agrawal 2010 (180)  Hunter 2016 (564)  Lees 2018 (361)  Yagi 2023 (67) |
| **Radiographic factors** | | | |
| **Larger tumour size** | | Paldor et al, 2016 (772 patients across 7 studies in review)  Daultrey 2016 (900)  Hunter 2016 (564)  Joo 2017 (97)  Younes 2017 (53)  Borsetto 2018 (112)  D’Haese 2019 (62)  Fieux 2020 (336)  Hentschel 2020 (1217)  Schnurman 2020 (212)  Kim 2021 (118)  Marinelli 2022 (952)  Stastna 2024 (615) | Herwadkar 2005 (50)  Whitehouse 2010 (88)  Paldor et al, 2016 (1557 patients across 13 included studies in review)  Kleijwegt 2020 (738)  Higuchi 2021 (53)  Itoyama 2022 (64)  Yagi 2023 (67)  Marinelli 2023 (405) |
| **Extracanalicular VS at presentation** | | Hajioff 2008 (72)  Hughes 2011 (59)  Daultrey 2016 (900)  Wolbers 2016 (155)  Younes 2017 (53)  Borsetto 2018 (112)  Lees 2018 (361)  Fieux 2020 (336)  Kleijwegt 2020 (738)  Hentschel 2020 (1217)  Kim 2021 (118)  Higuchi 2021 (53)  Sethi 2021(340)  Reznitsky 2021 (2312)  Yamada 2022 (31)  Stastna 2024 (615) | Flint 2005 (100)  Godefroy,2009 (70)  Agrawal 2010 (180)  Timmer 2011 (240)  Patnaik 2015 (576)  van de Langenberg 2011 (36)  Hunter et al 2016 (564)  Prasad 2018 (576)  D’Haese 2019 (62)  Yagi 2023 (67)  Moffat 2012 (381, *found that tumours located in the IAC had a faster growth rate compared to those located in the CPA at time of diagnosis) |
| **Growth within the first year** | | Tschudi 2000 (74)  Flint 2005 (100)  Stangerup 2006 (552)  Whitehouse 2010 (88)  Eljamel 2011 (53)  van de Langenberg 2011 (36)  Sethi 2019 (341)  Marinelli 2021 (592)  Marinelli 2022 (952)  Stastna 2024 (615) | D’Haese 2019 (62) |
| **Cystic VS** | | Charabi 1994 (23)  Selesnick 1998 (571)  Piccirillo 2009( 57)  Varughese 2012 (178)  Han 2018 (220)  Kleijwegt 2020 (738)  Hentschel 2020 (1217)  Itoyama 2022 (64) (*based on radiomic features)  Stastna 2022 (125)  Stastna 2024 (615) | Tomita 2015 (43)  Higuchi 2021 (53)  Kim 2021 (118)  Yamada 2022 (31) |

**Supplementary table S2: Studies evaluating radiomic based analyses for evaluating radiosurgery response**

| **Author** | **Study design** | **N** | **Follow-up period** | **Definition of treatment response/ failure** | **Sequences and analysis used** | **Key findings** | **Potential limitations** |
| --- | --- | --- | --- | --- | --- | --- | --- |
| **D’amico et al 2018** | *Retrospective* | 38 | 10 years | Not defined. | Contrast enhanced T1-weighted images, 1.5T.  1135 shape-based, intensity-based and texture-based features. | 85.33% accuracy could be achieved to differentiate patients with tumour volume changes at 10 years compared to no volume change or volume increase. | Treatment response/treatment failure not defined.  Pseudoprogression not considered. |
| **Narayanasamy et al, 2019** | *Retrospective* | 32 | Not defined | Treatment failure if tumour volume increased > 10%. | T2-weighted imaging, 1.5T.  55 radiomic features extracted including: intensity, fractals, Laplacian of Gaussian and textural co-occurrence, run-length (RL), size Zone, and neighbourhood grey-tone difference matrices (NGTDM) features | Complexity in NGTDM (Neighbourhood Grey-Tone Difference matrices) and run percentage in RL (run-length), displayed AUC > 0.65 for prediction of treatment failure. | Follow up duration not defined.  Pseudoprogression not considered. |
| **Speckter et al, 2019** | *Retrospective* | 23 | Mean FU of 42.7 months (range 23.7–80.3 months). | Early transient progression defined as growth ≤18 months after treatment.  Later progression defined as growth > 18 months after treatment. | T1-weighted, T2-weighted , FLAIR and contrast enhanced T1-weighted imaging, 3T.  Signal intensity values were normalised to white matter values.  Texture features: mean value, standard deviation, minimum and maximum, skewness and kurtosis, and 2.5 and 97.5 percentile were used. | Lower quartile of kurtosis of relative T2-weighted image intensity values predicted any progression (transient or permanent) with a sensitivity and specificity of 71% and 78%.  Only the minimum of the normalized T2-weighted image intensity values correlated significantly to the final reduction of tumour volume per month. | No clear clearly defined volumetric threshold for defining progression/ response.  Mixing of transient and later progression in deriving sensitivity measures of kurtosis. |
| **Langenhuizen et al, 2020** | *Retrospective* | *99* | All patients had an MRI scan at 6 months following treatment and were followed‐up for at least 18 months. | Transient tumour enlargement (TTE) = volumetric increase of ≥10% within the first 12 months after treatment, followed by volumetric reduction to at least the tumour volume at treatment.  *Threshold for volumetric increase chosen based on inter‐ and intra‐observer variability analysis of the tumour. | T1-weighted, T2-weighted, and contrast-enhanced T1-weighted imaging.  Image features extracted: first-order statistics, Minkowski functionals (MFs), and three-dimensional grey-level co-occurrence matrices (GLCMs). | Set of 4 GLCM features, achieved a sensitivity of 0.82 and a specificity of 0.69 of predicting TTE.  Greater prediction of TTE for larger tumour volumes obtaining a sensitivity of 0.77 and a specificity of 0.89 for tumours larger than 6 cm^3^. | Retrospective study.  Differences in MRI scanner protocol and inter-subject signal intensity variations.  Lack of histological validation for distinguishing TTE from true tumour growth.  Tumours less than 1.42 cm^3^ in size were not included. |
| **Langenhuizen et al, 2020** | *Retrospective* | 85 | All tumours had at least 2 years FU post treatment  Latest-occurring failure at 129 months | Tumour control = No progression after 129 months. (*Based on latest-occurring failure at 129 months).  Tumour progression = Volumetric progression beyond 2 years after treatment.  Growth = Significant increases (>10% in tumour volume among three consecutive follow-up MRI). | T1-weighted, T2-weighted, and contrast-enhanced T1-weighted imaging.  After normalization, the following radiomic features were extracted.  1) Twenty first-order statistics (FOS) features  2) Four Minkowski functionals (MFs) ^(^  3) Four grey-level co-occurrence matrix (GLCM)  4) Thirteen grey-level size zone matrix (GLSZM) features | Grey-level co-occurrence matrices had 0.77 accuracy, 0.71 sensitivity, 0.83 specificity, and 0.93 AUC for predicting long term tumour control.  Prediction scores improved to 0.83, 0.83, 0.82, and 0.99, respectively, for tumours larger than 5 cm^3^. | Retrospective study with usage of MRI data > 10 years old for tumour texture analysis.  MRI field strength not defined.  Single-centre data.  Possible overfitting of trained models for larger tumours.  Tumours less than 1.42 cm^3^ in size were not included. |
| **George-Jones et al, 2021** | *Retrospective* | 53 | Median follow-up of 6.5 months (interquartile range/IQR, 5.9-7.4) | Post-SRS enlargement >20% of the pre-treatment volume. | Contrast-enhanced T1-weighted imaging  Texture and shape features from the SRS planning scans were extracted and used to train a linear support vector machine.  Thirteen parameters were related to the shape of the tumour, 18 parameters were related to a first-order analysis of voxel intensities, and 24 parameters were derived from grey-level co-occurrence matrices (GLCM). | Model had a sensitivity of 92%, specificity of 65%, AUC of 0.75, and a positive likelihood ratio of 2.6 (95% CI 1.4-5.0) for predicting post-SRS enlargement of >20%. In the larger tumour subgroup, the model had a sensitivity of 87%, specificity of 73%, AUC of 0.76, and a positive likelihood ratio of 3.2 (95% CI 1.2-8.5). | Small sample size.  MRI field strength not defined.  Short follow up period. |
| **Yang et al, 2021** | *Retrospective* | 336 | Median follow-up of 65.1 months (at least 2 years for each patient) after radiosurgery | Tumour regression = Reduction of tumour volume ≥10%, comparing the final volume to the pre-SRS volume.  Non-response = Volume change <10% or tumour enlargement. | T1-weighted, T2-weighted, and contrast-enhanced T1-weighted imaging, 1.5T.  1736 MR radiomic features were generated for each patient. | Five radiomic features associated with the inhomogeneous hypointensity pattern of contrast enhancement and the variation of T2-weighted intensity achieved:  Prediction of long-term outcome achieved an accuracy of 88.4%  Prediction of transient pseudoprogression achieved an accuracy of 85.0% | Large slice thickness (>3mm) may have impacted on accuracy of tumour volumetric measurements.  Single-centre study. |
| **Bossi-Zanetti et al 2023** | *Retrospective* | 108 | Up to 36 months | Volume response defined at 24 months and 36 months.  An increased tumour volume was observed at 24 months in 12 patients, and at 36 months in another group of 12 patients. | Contrast-enhanced T1-weighted imaging acquired at 2 centres, 1.5T.  Following classes of features were extracted: shape (*n* = 14), first-order (*n* = 18), grey-level co-occurrence matrix (GLCM, *n* = 24), grey-level dependence matrix (GLDM, *n* = 14), grey-level run length. matrix (GLRLM, *n* = 16), grey-level size zone matrix (GLSZM, *n* = 16), and neighbouring grey tone difference matrix (NGTDM, *n* = 5).  Four ML algorithms (Random Forest, Support Vector Machine, Neural Network, and extreme Gradient Boosting) were trained and tested for treatment response | The neural network was the best predictive algorithm for response at 24 (balanced accuracy 73% ± 18%, specificity 85% ± 12%, sensitivity 60% ± 42%) and 36 months (balanced accuracy 65% ± 12%, specificity 83% ± 9%, sensitivity 47% ± 27%). | Retrospective design so possible changes in MRI protocols over time.  No clear clearly defined volumetric threshold for defining progression/ response.  Analysed only T1-weighted images with contrast enhancement.  Lack of volume size stratification.  Different cyberknife and dosing protocols. |

**Supplementary table S3: Studies adopting use of DCE-MRI for evaluating radiosurgery response**

| **Author** | **Study design** | **N** | **Follow-up period** | **Definition of treatment response/ failure** | **Key findings** | **Limitations** |
| --- | --- | --- | --- | --- | --- | --- |
| **Lewis et al, 2021** | *Prospective* | 5 | Multimodal MRI imaging up to 6 months.  ^23^Na-MRI and DCE-MRI were obtained from all patients before radiosurgery, at 2 weeks, 8 weeks and 6 months post-treatment. | Mechanistic study.  Response and failure not defined. | Changes in tumour total sodium concentration (TSC) and microvascular parameters (K^trans,^ v_p_, v_e_) were observable 2 weeks post-treatment, preceding changes in structural imaging.  At 6 months post-treatment there were significant voxelwise increases in tumour TSC and mean diffusivity with decreases in tumour microvascular parameters (K^trans^, v_e_, v_p_). | Pilot mechanistic study so didn’t evaluate for predictors of treatment response/ failure beyond 3 years  Small patient number. |
| **Ozer et al, 2022** | *Prospective* | 24 | 56 months (range 48 to 59 months).  DCE-MRI sequences were obtained from all patients before radiosurgery, and at 3 and 6 months. | Change of 10% in tumour volume was considered significant  Changes in tumour volume were classified as regression or stable (RS), transient tumour enlargement (TTE), and continuous tumour enlargement (CTE). TTE and CTE were differentiated based on whether there was shrinkage after tumour growth following radiosurgery. | Patients with regression or only transient tumour enlargement demonstrated lower K^trans^ and v_e_ values at 3 and 6 months after radiosurgery compared to pre-treatment  *K*^trans^ and v_e_ parameters at 3 and 6 months provided excellent diagnostic performance in predicting treatment response following radiosurgery (AUC > 0.9). | Small sample size.  Single centre study.  Use of low temporal resolution (Δt = 15 seconds) DCE-MRI sequence.  Plasma volume not considered because the Toft and Kermode kinetic analysis method rather than the extended Tofts model was used. |
| **Meng et al, 2024** | *Prospective* | 19 | Median follow-up time was 56.4 months.  Dynamic GRASP studies were acquired before SRS, as well as at 6, 18, and approximately 30 months post-treatment. | Response and failure not defined. | At 6 months, all semi-quantitative parameters (peak, AUC, wash-in slope), were significantly reduced relative to pre-treatment.  Correlation between the reduction in peak, AUC and wash-in slope at 6 months and % tumour volume reduction beyond 4 years. | Only cases with initial loss and then restoration of central homogenous contrast enhancement on postcontrast T1-weighted MRI were included.  No comparison between tumours with long-term regression, stability, or progression after SRS.  Small sample size.  Use of semi-quantitative parameters only. |
| **Hwang et al, 2022** | *Prospective* | 35 | Mean interval between SRS and follow-up imaging was 30.2 ± 5.7 months  Mean follow-up in responders 30.9 ± 5.3 months.  Mean follow-up in non-responders 29.0 ± 6.4 months. | Response defined as tumour volume reduced by more than ≥ 20% on the last follow-up MRI. | Responding tumours demonstrated significantly lower mean pre-treatment tumour K^trans^ and area under the DCE curve of the initial 90-s postcontrast agent arrival (IAUC_90_).  *K*^trans^ and IAUC_90_ demonstrated a sensitivity and specificity of 81.8% / 100% and 69.2% / 53.8% respectively for tumour response prediction | Follow up period for assessing response post SRS was less than 3 years.  Pre-treatment growth rate was not quantified. |
